# Distinct Molecular Patterns of Two-Component Signal Transduction Systems in Thermophilic Cyanobacteria as Revealed by Genomic Identification

**DOI:** 10.3390/biology12020271

**Published:** 2023-02-08

**Authors:** Jie Tang, Dan Yao, Huizhen Zhou, Mingcheng Wang, Maurycy Daroch

**Affiliations:** 1 School of Food and Bioengineering, Chengdu University, Chengdu 610106, China; 2 School of Environment and Energy, Peking University Shenzhen Graduate School, 2199 Lishui Road, Shenzhen 518055, China

**Keywords:** thermophilic cyanobacterium, histidine kinase, two-component systems, signal transduction

## Abstract

**Simple Summary:**

Although the two-component system is known to play considerable roles in sensing and responding to environmental signals, there is little information regarding the two-component systems of thermophilic cyanobacteria. Herein, we investigated the structure and architecture of two-component systems in 17 well-described thermophilic cyanobacteria. The results revealed a fascinating complexity and diversity of these systems. Moreover, the distinct composition of genes related to these systems existed among these thermophilic cyanobacteria. In addition, we found diversified domain architectures of histidine kinases and response regulators, putatively in association with various functions. Furthermore, horizontal gene transfer, as well as duplications events, might be involved in the evolutionary history of genes relevant to these systems in certain genera. The obtained data will highlight that the genomes of thermophilic cyanobacteria have a broad potential for acclimations to environmental fluctuations.

**Abstract:**

Two-component systems (TCSs) play crucial roles in sensing and responding to environmental signals, facilitating the acclimation of cyanobacteria to hostile niches. To date, there is limited information on the TCSs of thermophilic cyanobacteria. Here, genome-based approaches were used to gain insights into the structure and architecture of the TCS in 17 well-described thermophilic cyanobacteria, namely strains from the genus *Leptodesmis*, *Leptolyngbya*, *Leptothermofonsia*, *Thermoleptolyngbya*, *Thermostichus*, and *Thermosynechococcus*. The results revealed a fascinating complexity and diversity of the TCSs. A distinct composition of TCS genes existed among these thermophilic cyanobacteria. A majority of TCS genes were classified as orphan, followed by the paired and complex cluster. A high proportion of histidine kinases (HKs) were predicted to be cytosolic subcellular localizations. Further analyses suggested diversified domain architectures of HK and response regulators (RRs), putatively in association with various functions. Comparative and evolutionary genomic analyses indicated that the horizontal gene transfer, as well as duplications events, might be involved in the evolutionary history of TCS genes in *Thermostichus* and *Thermosynechococcus* strains. A comparative analysis between thermophilic and mesophilic cyanobacteria indicated that one HK cluster and one RR cluster were uniquely shared by all the thermophilic cyanobacteria studied, while two HK clusters and one RR cluster were common to all the filamentous thermophilic cyanobacteria. These results suggested that these thermophile-unique clusters may be related to thermal characters and morphology. Collectively, this study shed light on the TCSs of thermophilic cyanobacteria, which may confer the necessary regulatory flexibility; these findings highlight that the genomes of thermophilic cyanobacteria have a broad potential for acclimations to environmental fluctuations.

## 1. Introduction

Cyanobacteria are a very large and morphologically diverse group of oxygen-evolving photosynthetic prokaryotes. They show a cosmopolitan distribution in most terrestrial, freshwater, and marine habitats, and even in extreme environments, from Antarctica to hot springs [1,2,3]. Cyanobacteria account for approximately 40% of the planetary oxygen production [4]. More intriguingly, cyanobacteria are considered to be a promising candidate for mitigating CO_2_ in the context of global warming and greenhouse gas emissions [5]. However, to fully explore the industrial potential of cyanobacteria requires thorough studies on each biological block of this organism.

As in most prokaryotes, cyanobacteria utilize two-component systems (TCSs) as some of the major pathways to sense and coordinate their behavior in response to changes in the external environment [6,7,8]. TCS play considerable roles in an extensive spectrum of adaptive mechanisms, such as chemotaxis, metabolism, motility, etc. [9]. A typical TCS comprises two types of proteins, histidine kinases (HKs) and cognate response regulators (RRs) [10]. HKs are usually membrane-bound and are characterized by the presence of specific signatures: a HisKA (dimerization and phosphoacceptor) and a HATPase domain (histidine kinase ATPase), while RR contains a receiver domain (REC) [11]. According to the prevailing program, upon sensing a stimulus on the input domain of the N-terminal variable region, the HisKA and HATPase domains of the HK function by autophosphorylating a conserved histidine residue and subsequently transferring the phosphate group to a conserved aspartyl residue at the REC of the RR. Phosphorylating the RR results in the activation of the downstream output domain that leads to specific responses. Additionally, HK fused to REC are designated as hybrid HKs (hyHKs), which afford numerous intramolecular phosphotransfer reactions and/or phosphorelays [12].

The increasing number of prokaryotes with complete genome sequences has dramatically extended our cognition of TCS prevalence among prokaryotes [13]. Moreover, these insights into the TCS revealed great variations of the TCS gene number among organisms and a firm correlation between the ecological niche and sophistication of behaviors in prokaryotes [14,15]. In recent years, next-generation sequencing (NGS) has been widely used to elucidate cyanobacterial genomes in thermal environments [16,17]. Although the commonly conserved two-component systems have already been elucidated in cyanobacteria [6], an evolution under different habitats may lead to the extant repertoire of TCS genes with the help of the occurrence of duplications, fusions, gene losses, insertions, and deletions, as well as domain shuffling. Nevertheless, very little is known about the TCS genes in thermophilic cyanobacteria.

It has been reported that the utilization of the TCS in the construction of synthetic genetic networks may be useful for engineering novel cell functions, such as biofuel-related metabolic engineering in microorganisms by the implementation of genetic biosensors [18]. In addition, autotrophic cyanobacteria is likely to have a different regulatory mechanism of the TCS due to the trophic types [12]. Moreover, thermophilic cyanobacteria might evolve into a unique TCS in light of the strong selective pressure caused by hostile habitats. Understanding the sequences, domain architectures, and biology of the TCS genes in thermophilic cyanobacteria will benefit as a prerequisite for clarifying their exact biochemical functions by integrating genomics, proteomics, and metabolomics data [19,20]. Furthermore, the contextual information of TCS gene organization can be very vital, providing clues regarding the signaling pathway structure (HK-RR partnerships) and the TCS function [21].

In the current work, we analyzed the TCS repertoire of thermophilic cyanobacteria. The distribution and genetic/domain organization of the TCS genes were assessed. Insights were further gained for the structure and architecture of HKs and RRs. Moreover, the conserved TCS genes were identified, and comparative and evolutionary genomic analysis revealed the importance of accessory TCS genes in the expansion of TCS genes through horizontal gene transfer (HGT) and gene duplication events. In addition, we hypothesized that a unique TCS may be contained by thermophiles due to their thermal characters.

## 2. Materials and Methods

### 2.1. Genome Collection of Thermophilic Cyanobacteria

A dataset of 17 thermophilic cyanobacteria was retrieved from the genomic resources of the NCBI. The collection of these strains was established according to our previous study through performing a filtration based on NCBI genomic resources, validation of thermophilic characteristics, and quality control of genomic sequences [22]. Detailed information regarding morphology, ecology and genome characteristics of the 17 thermophiles was compiled in Table S1. In brief, the 17 thermophiles were taxonomically affiliated to four families of the order Pseudanabaenales and Synechococcales, including Leptolyngbyaceae: *Leptodesmis sichuanensis* A121 [23], *Leptolyngbya* sp. JSC-1 [24] and *Leptothermofonsia sichuanensis* E412 [25]; Oculatellaceae: *Thermoleptolyngbya* sp. O-77 [26] and *T. sichuanensis* A183 [27]; Synechococcaceae: *Thermostichus* sp. 60AY4M2, 63AY4M2, 65AY6A5, 65AY6Li [28], JA-2-3B and JA-3-3Ab [29]; Thermosynechococcaceae: *Thermosynechococcus lividus* PCC 6715 [22], *Thermosynechococcus* sp. CL-1 [30] and TA-1 [31], *T. vestitus* BP-1 [32] and E542 [33], *T. vulcanus* NIES-2134 [34].

### 2.2. Identification of TCS Genes

The TCS genes in each genome were identified using P2RP (http://www.p2rp.org/, accessed on 15 May 2022) [35]. All TCS genes detected were categorized as HK, RR or PP (phosphotransfer protein). HKs were further grouped into classic (cHK) or hybrid (hyHK) based on the presence of a REC domain within the protein, and Che-like or unorthodox HK. TCS genes classified by P2RP as ‘Incomplete HK’ were manually validated based on the following criteria: (i) the presence of both HisKA domain and HATPase; (ii) exceptions were made for HKs whose transmitter region was composed solely of a HisKA domain but its gene was adjacent to that encoding another TCS gene [36], while ‘mispredicted’ TCS proteins were excluded from the analysis. All the putative TCS genes identified were subject to a manual curation to avoid any biased results. The Pearson coefficient was employed to assess the correlation between the TCS gene number and genome size by using the cor.test function in R v3.6.2. Significance levels of 0.05 and 0.01 were applied for the analysis.

According to the genetic organization on the DNA, TCS genes were classified as orphan when no TCS gene was adjacent to another TCS gene, as paired when two TCS genes were adjacent, and as complex when more than two genes were contiguous [37]. Given functional reasons [37], the definition of a gene cluster (paired or complex) was further validated by two parameters: (1) intergenic distances within a cluster had to be less than 200 bp; (2) genes had to lie in the same transcription direction or in a divergent direction on the DNA.

### 2.3. Protein Sequences Analysis

The protein sequences of TCS genes were downloaded from results calculated by P2RP. Domains within protein sequences of TCS genes were identified by the PFAM tool of European Bioinformatics Institute (EMBL-EBI) (http://pfam-legacy.xfam.org/, accessed on 20 June 2022). Protein alignments were performed with the default parameters using Muscle implemented in Mega7 [38]. For the secondary structure prediction, the protein sequences were analyzed with the program Geneious [39].

### 2.4. Identification of Orthologous Proteins

According to the bidirectional best hit (BBH) criterion, orthologous proteins of TCS genes in focal taxa were identified using BLASTP. The BLASTP alignments were performed with the following thresholds: identity percentage greater than 40% and query coverage greater than 75%.

### 2.5. Phylogeny

Maximum-likelihood (ML) phylogenetic analyses of HKs and RRs were carried out using PhyML v3.0 [40], and the substitution models were selected by the Model Selection function implemented in PhyML [41] under the Akaike information criterion (AIC). Parameter settings in PhyML and bootstrap analysis of phylogenies were followed as described [42]. Tree topologies were visualized and drawn using iTOL V5 [43]. Moreover, the similarity clustering was built using an unweighted pair group method with arithmetic means (UPGMA) based on the ANI (Average Nucleotide Identity) matrix estimating all-against-all distances in a collection of focal genomes [44] and visualized using Mega7.

## 3. Results and Discussion

### 3.1. Composition of TCS Genes in Thermophilic Cyanobacteria

The general features of predicted TCS genes in the genomes of 17 thermophilic cyanobacteria were summarized in Table 1, and detailed results were presented in Table S2. These genomes possessed a different number of putative TCS genes, ranging from 42 (*Thermosynechococcus* NIES-2134) to 239 (*Leptolyngbya* JSC-1). Intergenic variations were evident in TCS gene number (Table 1), whereas there were limited intragenus variations. Similar patterns were observed in the gene number of HK and RR. Few PPs (standalone HisKA or Hpt domain-containing proteins) were identified in each genome. However, the number of PPs identified in this study may be underestimated in that the low sequence conservation often hindered the Hpt domain detection [45]. In addition, a very small proportion of ORFs in each genome was devoted to TCS genes, varying from 1.7% to 3.2%. Within the category of HK, cHK and hyHK were the most abundant types, accounting for a vast majority of HK identified in each genome. The ratio of the receiver (REC domains possessed by RRs and hyHKs) and transmitter (HisKA and HATPase domains contained by cHKs and hyHKs) demonstrated considerable intergenic fluctuations. The receiver/transmitter ratios for *Leptodesmis*, *Leptolyngbya*, *Leptothermofonsia*, *Thermostichus* and *Thermosynechococcus* were 1.3, 1.4, 1.2, 1.6–2.0 and 1.8–3.2, respectively. Particularly, *Thermosynechococcus* PCC6715 demonstrated a receiver/transmitter ratio of 3.2, which was much higher than that (1.8–2.2) of the other *Thermosynechococcus* strains. The result indicated that one HK might phosphorylate more than one RR in these thermophilic cyanobacteria. On the contrary, the *Thermoleptolyngbya* strains demonstrated a receiver/transmitter ratio of 0.9, suggesting that one RR may be phosphorylated by more than one HK. Inconsistent receiver/transmitter ratios were also observed in other cyanobacterial genomes (Table S3). Although it is well-known that the canonical RR are often in operons with the HK, the above results indicate that phosphorylations between non-cognate HK-RR pairs are normal in cyanobacteria as responses to various stresses by the cross-talk in signal transduction that is taking place among two-component systems [46].

**Figure 1:**
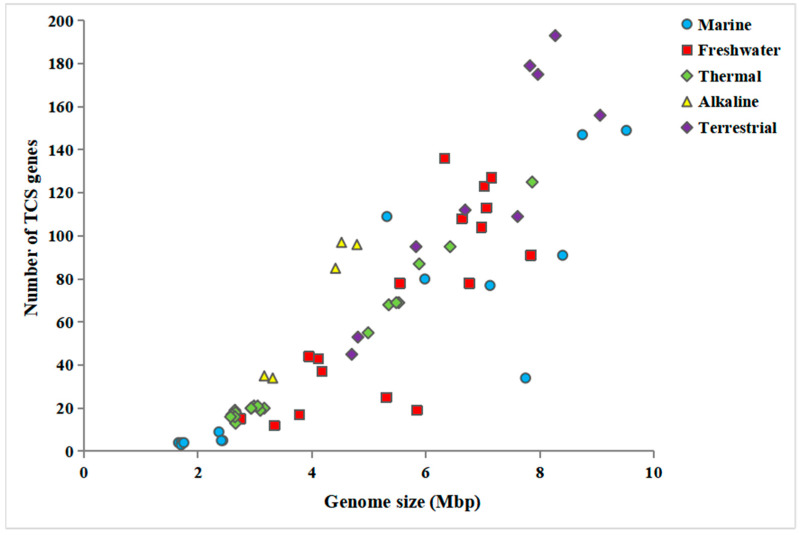
Correlation between TCS gene number and genome size.

Intriguingly, the TCS gene number appeared to be positively associated with genome size (Table 1). To verify this speculation, more cyanobacterial genomes on a larger scale were collected for the correlation analysis. Overall, we compiled a genome dataset of 64 cyanobacterial genomes (Table S3). The dataset represented a diverse array of cyanobacteria from five ecological niches, including marine, freshwater, thermal, alkaline and terrestrial niches. As shown in Figure 1, the number of TCS per genome was demonstrated to be positively correlated with genome size, although several outliers deviated from the trend. This result was further verified by a positively significant correlation (ρ = 0.88, *P* < 0.01), as suggested by the Pearson analysis.

Additionally, there is one thing to bear in mind. Previous studies strongly suggested that *Leptolyngbya* sp. JSC-1 was not a phylogenetic member of the genus *Leptolyngbya sensu stricto* but represented a new genus within the family of Leptolyngbyaceae [27,47]. Nevertheless, the actual taxonomy of JSC-1 has not yet been validated and *Leptolyngbya* sp. is not a valid name for this strain.

### 3.2. Genetic Organization of TCS Genes in Thermophilic Cyanobacteria

From the perspective of function, genes inside the same operon often function in the same pathway. Thus, thoroughly understanding the genetic organization of TCS genes is crucial information so as to provide a potential TCS partnership. Herein, we investigated the genetic architecture of TCS genes in genomes of the 17 thermophilic cyanobacteria (Table S2). A majority of TCS genes were classified as orphan, ranging from 57.3% to 90.2% in the TCS genes of each genome, whereas paired TCS genes varied from 3.9% to 29.8% (Table 2). In addition, a small proportion (<13.5%) of TCS genes were organized in a complex cluster. Except for PCC6715, no TCS complex was found in *Thermosynechococcus* genomes. Among these complexes, the number and loci organization varied among the thermophilic strains (Table 2 and Table S2). Most of the complexes were a triad, while a tetrad was only detected in the *Leptothermofonsia* genome (RR-RR-cHK-RR) and Pentad in the genomes of *Leptolyngbya* (RR-cHK-RR-RR-hyHK), *Leptothermofonsia* (hyHK-RR-hyHK-hyHK-RR) and *Thermoleptolyngbya* (RR-RR-hyHK-hyHK-hyHK). Nevertheless, the putative phosphotransfer routes deduced from the configurations of these complexes were difficult to comprehend and will require further experimental verification.

In contrast to the genetic organization of TCS genes in thermophilic cyanobacteria, the TCS genes in *Escherichia coli* were primarily organized as pairs (71.5%), followed by the orphaned (14.5%) and complex (14.5%) [45]. By comparing cyanobacteria with similar genome sizes, the genetic organizations of TCS genes in thermophilic cyanobacteria showed slight differences with those of the corresponding reference cyanobacteria (Table 2), while on the whole, the organizations of the complex were more variable than the orphaned and paired.

Interestingly, apart from the conventional genetic organization of TCS genes (orphan or paired), the TCS complexes tend to be enriched in cyanobacteria with larger genomes (Table 2). This observation was in accordance with the prevailing concept that as genome size increased, cross-talk among TCS proteins may be beneficial for complicated signal transduction pathways [48]. Taken together, this implied that some thermophiles herein may have evolved complex signaling transduction pathways that allow a superior acclimation to a specific niche.

### 3.3. Cellular Localization of HK in Thermophilic Cyanobacteria

**Figure 2:**
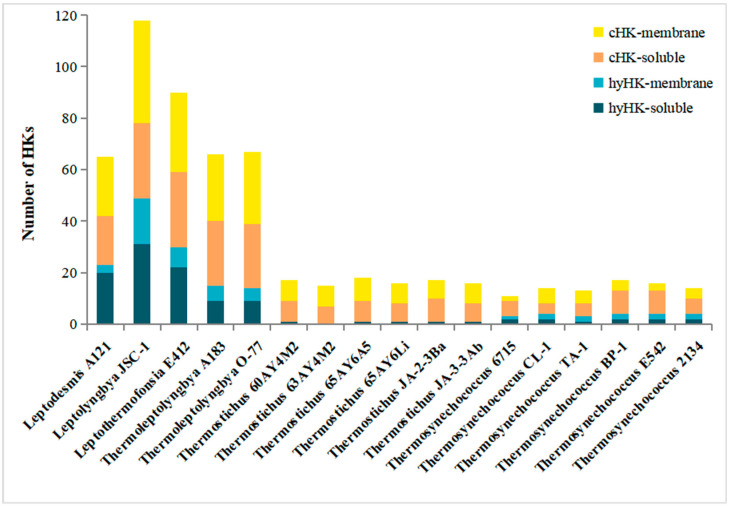
Predicted subcellular localization of HKs in the thermophilic cyanobacteria studied.

Herein, we specifically focused on TCS proteins that were not a part of Che-like or unorthodox systems. Thus, the HK afterwards referred to cHK and hyHK. The existence of transmembrane helices was used to evaluate the cellular localization of each HK identified. In the 17 thermophilic cyanobacteria, 42.9% to 72.7% of HKs were predicted to comprise no transmembrane segment (Figure 2), indicating cytosolic subcellular localization. At the intragenus level, proportions of the soluble HKs tremendously varied in *Thermostichus* (46.7–58.8%) and *Thermosynechococcus* (42.9% to 72.7%). The proportion of the soluble HKs observed in thermophilic cyanobacteria was comparable to that of other cyanobacteria according to the TCS database (http://www.p2cs.org/, accessed on 20 August 2022), e.g., *Nostoc punctiforme* PCC 73102 (65%) and *Prochlorococcus marinus* str. MIT 9313 (40%). Initially, soluble HKs were considered to sense intracellular signals (e.g., ATP, redox potential, reactive oxygen species), supervising the internal metabolic status of bacteria [49]. Notwithstanding this function, soluble HKs could also perceive membrane-diffusible signals. As a result, these systems could be responsible for a wide range of physicochemical parameters in the complex ecosystem of thermal habitats. Interestingly, the majority of hyHKs (>60%) (Figure 2) were predicted to be soluble in the genomes of *Leptodesmis*, *Leptolyngbya*, *Leptothermofonsia*, *Thermoleptolyngbya* and *Thermosynechococcus* PCC6715. Such a prevalence of cytosolic hyHKs may provide a selective advantage for an organism through facilitating spatial proximity with the corresponding cognate RR, consequently improving the velocity and efficiency of the signaling route [50].

### 3.4. Sensing-Domain Architecture of HKs in Thermophilic Cyanobacteria

Sensing domains are responsible for sensing environmental signals and are important to comprehend the essence of the signals perceived by individual HK [51]. In the HKs of surveyed thermophilic cyanobacteria, a proportion of 16.8% to 38.1% was found to contain no sensing domain (Table S2), indicating that these HKs may consist of either unknown sensing domains, or, they, as auxiliary proteins, may be affiliated to other complex signaling systems [52]. The sensing domains varied among the thermophilic cyanobacteria in terms of number and nature (Table 3).

The most abundant sensing-domain within HKs was the PAS (Per-Arnt-Sim) domain, followed by the GAF (cGMP-specific phosphodiesterases, Adenylyl cyclases and FhlA) domain and HAMP (Histidine kinases, Adenylyl cyclases, Methyl-binding proteins and Phosphatases) domain (Table 3). However, an exception was observed in *Thermosynechococcus* strains, which followed the pattern: GAF > PAS > HAMP domain. In addition, only in *Leptolyngbya* JSC-1 PAS domains appeared to be more abundant in hyHKs than in cHKs (61 versus 31), whereas the opposite trend was observed in the other 16 strains. PAS domains mainly function as monitoring changes in extracellular or intracellular environments by sensing chemical or physical signals, e.g., small molecules, light, redox state, ions and gases [53]. The presence of multiple homologous PAS domains in a single HK (Table S2) indicated that the HK could sense diverse signals. The GAF domains structurally share a high homology with the PAS domains, and they function as sensors of redox, oxygen or as cGMP binding [54]. The HAMP domains are essential for signal transduction [55], though they are not recognized to sense signals. The main function of the HAMP is assumed to be in converting signals from the TM helices to those that can be recognized by the downstream cytoplasmic domains.

**Figure 3:**
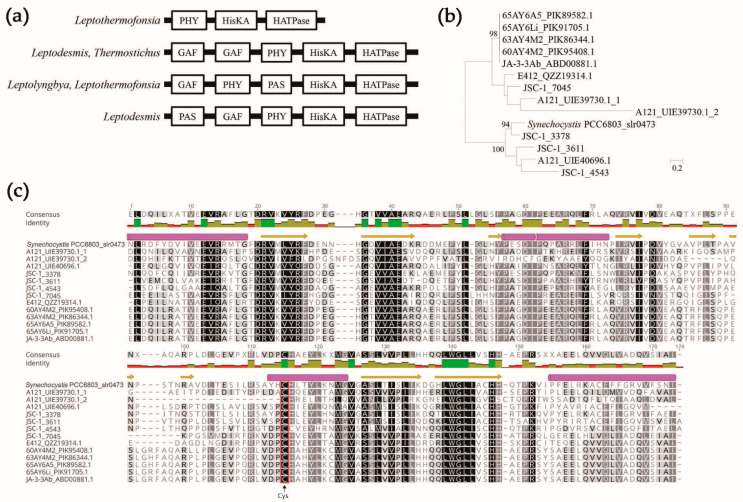
Molecular characterization of PHY-containing HKs. (**a**) Domain structure of PHY-containing HKs. (**b**) Phylogenetic inference of GAF domains within PHY-containing HKs. Scale bar = 20% substitutions per site. (**c**) Protein alignment of GAF domains within PHY-containing HKs. Chromophore-binding cysteine of Cys phytochromes were indicated by red box. The α-helix and β-sheet predicted were, respectively, indicated by cylinder and arrow.

At least one photoreceptor PHY (phytochromes-specific) domain was found only in *Leptodesmis*, *Leptolyngbya*, *Leptothermofonsia* and *Thermostichus* (except for strain JA-2-3Ba). All the PHY domains predicted were either standalone PHY (*Leptothermofonsia*) or in a cluster with other domains in the N-terminal of cHKs, forming different types of domain arrangements, namely PAS-GAF-PHY (*Leptodesmis*), GAF-GAF-PHY (*Leptodesmis*, *Thermostichus*) and GAF-PHY-PAS (*Leptolyngbya*, *Leptothermofonsia*) (Figure 3a). Phytochromes typically comprise N-terminal photosensory modules of PAS-GAF-PHY and a C-terminal domain involved in signal transduction, mediating sensory responses to the ambient light environment. In plant and cyanobacterial phytochromes, the GAF domain is necessary for the covalent attachment of photopigments (e.g., phytochromobilin and phycocyanobilin) by a conserved Cys residue [56]. A phylogenetic analysis indicated that the GAFs of the PHY-containing HKs demonstrated tremendous genetic diversity of the amino acid sequences, even within a single HK or individual strain (Figure 3b). A further structure analysis suggested that similar secondary structures of the GAFs were achieved (Figure 3c). Compared to the phytochrome (*slr0473*) from *Synechocystis* PCC 6803 [57], canonical Cys residue was present in all the GAFs of the PHY-containing HKs of thermophilic cyanobacteria (Figure 3c), suggesting fundamental functions in the physicochemical properties. Particularly, the PHY domain of phytochromes is a red/far-red photoreceptor in bacteria [58] and the photoacclimation of far-red light by *Leptolyngbya* JSC-1 and *Leptothermofonsia* E412 has been experimentally verified [25,59]. In addition, the diversity of phytochrome-like HKs among these thermophilic cyanobacteria that most likely reflect their specific in vivo functions warrants further study. Interestingly, no PHY domain was detected in the HKs of *Thermosynechococcus* strains, since they were typical cyanobacteria showing a blue/green-type reversible photoconversion. This should be ascribed to the cyanobacteriochromes, a group of photoreceptors distantly related to the phytochrome family [60]. Only the GAF domain is needed for the chromophore-binding region and proper photoconversion of the cyanobacteriochromes [61]. Although the GAF domains of cyanobacteriochromes are highly diverse and classified into many lineages, the green/blue lineage contains a conserved canonical Cys residue to covalently attach to the A ring of a linear tetrapyrrole chromophore and to stabilize chromophore incorporation [62]. Likewise, a conserved chromophore binding Cys is identified in the GAFs in *Thermosynechococcus* strains.

The remaining sensing domains showed an irregular occurrence, and some sensing domains were found to be specific to a few strains. For example, the cNMP binding, FHA and Pkinase domains were found exclusively in *Leptolyngbya*, *Thermosynechococcus* and *Leptolyngbya*. NIT (Nitrate- and nitrite- sensing) was only associated with *Leptothermofonsia* and *Thermoleptolyngbya*, suggesting that they may monitor the nitrate or nitrite concentration [21]. *Leptodesmis* and *Leptolyngbya* exhibited a Ser/Thr kinase catalytic domain (S_TKc), indicating that the two cyanobacteria may also have the eukaryotic-type signal transduction systems in addition to the prokaryotic two-component systems [63]. Taken together, thermophilic cyanobacteria might utilize a wide array of sensing strategies to acclimate to specific niches.

### 3.5. Domain Architecture of HKs in Thermophilic Cyanobacteria

Domain architecture is very informative for predicting the putative function of signaling proteins [64]. In the present study, we classified the domain architectures of HKs based on the number and order of their sensing domains (whatever the nature of the sensing domain), transmitter (comprising HisKA and HATPase domains), and receiver domains. In total, 43 distinct domain architecture combinations were found in the 17 thermophilic cyanobacteria (Table S4). As expected, thermophiles with more TCS genes showed more domain architecture combinations, ranging from 7 to 28 in each genome. Moreover, the hyHKs exhibited a wider range of domain combinations compared to their classical counterparts (35 versus 8), though the cHK accounted for the majority (64.6–93.3%) of HK domain architectures. Only *Leptolyngbya* JSC-1 demonstrated a median percentage (57.3%), which could be ascribed to the abundant hyHK domain architectures.

The top 10 abundant domain architectures are summarized in Table 4. Apart from the domain architecture without a sensing domain, typical domain architectures composed of sensing and transmitter domains were dominant. Given the limited hyHKs detected in *Thermostichus* and *Thermosynechococcus* genomes, the subsequent analysis focused on the hyHKs in *Leptodesmis*, *Leptolyngbya*, *Leptothermofonsia* and *Thermoleptolyngbya*. The hyHKs consisted of only one transmitter and one receiver domain was dominant among the hyHK domain architectures in the five genomes (71.4–77.6%), whereas the remaining hyHks showed atypical domain architectures with multiple transmitter and/or receiver domains. Those sophisticated domain architectures suggested that each protein may possess complex phosphotransfer pathways. Meanwhile, 24.5% to 50.0% of hyHKs in the five genomes contained no sensing domains, while the other hyHKs possessed multiple sensing domains: as many as 13 sensing domains (twelve tandem repeated HAMP-GAF, *Leptolyngbya* JSC-1) (Table S2). These proteins harboring multiple sensing domains may suggest their capability to perceive a wide range of signals. As for the hyHKs with two or more REC domains, they may have different roles in the phosphotransfer route, as suggested by in vitro phosphotransfer studies [65]. Nevertheless, the actual functions of these domains detected in this study merits further validation, e.g., by a mutant phenotype study.

### 3.6. Domain Architecture of RRs in Thermophilic Cyanobacteria

HKs can be easily identified due to the possession of a characterized transmitter domain, whereas RRs were defined by the possession of receiver domains [13]. However, a sequence similarity-based classification of RRs cannot facilitate the full determination of a putative biological function, whereas the output domain-based classification of RRs and architecture has been demonstrated to be useful in elucidating the putative RR functions [66]. According to the output domains, RRs identified in genomes of the 17 thermophilic cyanobacteria were classified into eleven RR families with known output domains (76.9–94.9%) and one with an unknown output domain (5.1–23.1%) (Table 5). The CheY-like and NarL/OmpR-like families were the major constituents of total RRs, accounting for 19.2–42.7% and 31.3–57.7%, respectively. The CheY-like RRs contained standalone REC domains but no output domain. In comparison, such RRs with standalone REC domains were also overrepresented in *N. punctiforme* PCC 73102 (36% of the total RRs) [6]. Initially, CheY-type RRs were characterized as chemotaxis regulation in *E. coli*, but their roles are far beyond as they are also able to act as connectors between TCS partners, thus facilitating crosstalk, feedback, and phosphorelays within the phosphorylation network of TCS [67]. As for NarL/OmpR-like RRs containing a DNA-binding domain, they may function as transcriptional regulators. No RNA-binding domain was found among RRs of thermophilic cyanobacteria, indicating that no regulation of gene expression through antitermination was mediated by RRs. In addition, enzymatic output domains were quite common among RRs of these thermophilic cyanobacteria, and they might be involved in the modulation of the secondary messenger c-di-GMP or Ser/Thr phosphorylation, suggesting the interconnection of TCS with other signal transduction pathways. The remaining RRs contained output domains that were unique to only one strain or several strains, implying that these strains may have more specific responses. However, the exact role of these domains in RRs needs further investigations.

### 3.7. Evolutionary Conserved and Accessory TCS in Thermostichus and Thermosynechococcus

According to the reciprocal best BLAST hit criterion, the most probable sets of orthologous TCS proteins shared by the *Thermostichus* or *Thermosynechococcus* strains were summarized in Table S5. The other thermophilic strains were excluded from this analysis due to the poor availability of genomes. For the six *Thermostichus* genomes, 222 (81.6%) out of 272 putative TCS genes were common to all genomes, revealing a strong conservation of TCS genes in *Thermostichus* strains. The core set of *Thermostichus* TCS genes included 72 cHKs and 150 RRs (Table S5). The ML phylogram showed that the core set of TCS genes was closely grouped in separate clusters (Figure S1), in accordance with the high similarities obtained by BLASTP alignments (Table S5). Among the conserved orthologous TCS genes, the overwhelming majority were genetically organized as orphans. Only one TCS pair was shared by the six *Thermostichus* strains, reflecting a possible co-evolution between the cognate HK and RR [68]. In addition, two strain-specific HK genes were identified only in *Thermostichus* JA-2-3Ba (Table S5).

**Figure 4:**
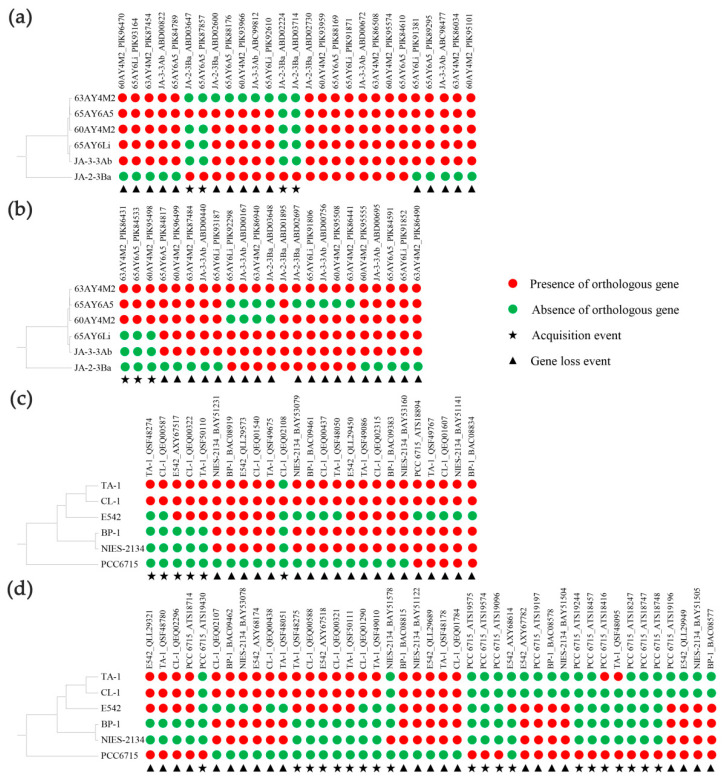
Occurrence of accessory TCS genes in *Thermostichus* (**a**: HKs, **b**: RRs) and *Thermosynechococcus* strains (**c**: HKs; **d**: RRs). The UPGMA trees on the left were built based on ANI distances of focal genomes. The HKs and RRs were named by the strain name plus accession number and shown in order of appearance in the respective phylograms (Figures S1 and S2).

As for the six *Thermosynechococcus* genomes, 174 (71.9%) out of 242 putative TCS genes were common to all genomes, including 48 cHKs, 12 hyHKs and 114 RRs (Table S5). However, no TCS pair among the conserved orthologous TCS genes was shared by the six *Thermosynechococcus* strains. The ML phylogram suggested that the core set of TCS genes were respectively closely clustered, and there were also some branches well separated (Figure S2). The BlastP analysis further verified 12 TCS genes (1 HK and 11 RRs) located in well-separated branches, as strain-specific genes (Table S5) may contribute to the niche-specific adaptation. The majority of strain-specific TCS genes were contributed by PCC 6715 (9 RRs), which was in accordance with the genome-based phylogeny of this species within the genus *Thermosynechococcus* (Figure 4).

### 3.8. Evolutionary Origin of Accessory TCS in Thermostichus and Thermosynechococcus

Compared to the core set of TCS genes, accessory TCS genes might contribute more to the genome plasticity. Herein, comparative and evolutionary genomic analyses were performed to reveal the origin of accessory TCS in *Thermostichus* and *Thermosynechococcus* strains. Among the 50 accessory TCS genes in the six *Thermostichus* strains, putative gene loss events were found in 34 TCS genes (Figure 4a,b). Moreover, most (20) of the possible gene lost events only occurred in JA-2-3Ba, indicating the possible recent loss events. The other gene loss events occurred either recently in a single *Thermostichus* strain or in a common ancestor of *Thermostichus* strains. Acquisition events were detected in seven TCS genes. Among them, two HKs (ABD02224 and ABD03714) were acquired only by JA-2-3Ba, suggesting that the two genes may have been recently acquired by this strain; the other two HKs (JA-2-3Ba _ABD03674 and 65AY6A5_PIK87857) might be independently acquired by the two strains; and the three RRs (63AY4M2_ PIK86431, 65AY6A5_PIK84533 and 60AY4M2_ PIK95498) might be acquired by the common ancestor of the three strains. All the events that occurred in JA-2-3Ba might contribute to the divergent genomes-based clustering between this strain and other *Thermostichus* strains (Figure 4a,b).

For the 68 accessory TCS genes in the six *Thermosynechococcus* strains, 42 gene loss events and 26 acquisition events were putatively detected (Figure 4c,d). A total of 15 gene loss events and 9 acquisition events occurred only in PCC 6715, in accordance with the phylogeny of this species among *Thermosynechococcus* strains (Figure 4c,d). Moreover, the two acquired RRs (ATS19574 and ATS19575) in PCC 6715 showed 100% identity (Table S5), indicating that the two RRs might have originated from a single acquisition event and then a duplication event happened. This result suggested that duplication events also played a crucial part in the expansion of TCS genes. The gene loss events in the other five strains happened recently in a single strain, a common ancestor of strains, or independently in two strains. Acquisition events in the other five strains happened only in the clade comprising CL-1, TA-1 and E542, including events in TA-1, in a common ancestor of TA-1 and CL-1, and in a common ancestor of TA-1, CL-1 and E542.

In summary, HGT, as well as duplication events, might be involved in the evolutionary history of TCS genes in *Thermostichus* and *Thermosynechococcus* strains. Specifically, our results were consistent with the previous report that numerous putative genes horizontally transferred from other bacteria have been actively acquired by *Thermosynechococcus* species, enabling the acclimation of them to stressful niches in hot springs [30]. Taken together, the results indicated genome plasticity and the expansion of TCS genes as coping with unique challenges that strains of the two genera faced.

### 3.9. Functional and Comparative Analysis of TCS between Thermophilic and Mesophilic Cyanobacteria

Typical, well-described, mesophilic cyanobacteria *Synechococcus* PCC 7942 and *Synechocystis* PCC 6803 were used as references to elucidate the phylogenetic relationship of TCS between thermophilic and mesophilic cyanobacteria. As shown in Figures S3 and S4, many HKs and RRs of thermophilic cyanobacteria, respectively, clustered with the HKs and RRs of mesophilic cyanobacteria, showing a close phylogenetic relationship. More intriguingly, it was ubiquitous in the phylograms that clusters were formed only by the HKs or RRs of thermophilic cyanobacteria. Taken together, these results suggested a generally conserved pattern of HKs and RRs shared by cyanobacteria and the characteristic molecular signatures in thermophiles. Among the clusters unique to thermophiles, only one HK cluster and one RR cluster appeared to be shared by all the thermophilic cyanobacteria studied. However, the HK of *Thermosynechococcus* PCC 6715 was missing in the shared HK cluster of thermophiles. The further BLASTP and domain analysis suggested that after the removal of one of the two fhlA domains, a pseudogene in the genome of PCC 6715 became highly homologous to the HKs of the shared cluster. The causes of the two duplicated fhlA domains in the pseudo gene are unknown. In addition, two HK clusters and one RR cluster were only common to all of the filamentous thermophilic cyanobacteria, suggesting that these HKs and RRs may be functionally related to the morphology. This could be indicative of the putative thermosensing function of histidine kinases present in this thermophile-specific clade [69]. The thermophile-unique clusters should be therefore experimentally validated in the near future.

The TCS in PCC 7942, Hik2 and Rre1, was reported to be highly conserved among cyanobacteria and to be a multi-stress responsive signal-transducing module that controls the expression of hspA, a chloroplast gene regarding redox regulation and other genes in cyanobacteria [69,70]. However, homologs of Hik2 were found in all the thermophilic cyanobacteria studied except for *Thermostichus* strains (Figure S3), showing low identities ranging from 33.2% to 36.4% to the Hik2 (Synpcc7942_453) in PCC 7942. Except for the *Thermosynechococcus* PCC 6715, all the thermophilic cyanobacteria contained homologs of Rre1 (Figure S4), exhibiting identities of 53.8–65.5% to that (Synpcc7942_1860) of PCC 7942. Rre1 was recently suggested to be the transcription factor responsible for the heat-shock-inducible transcription of major chaperone genes [71]. In addition, Rre1 can activate the genes encoding phycobilisomes and act as a negative regulator for salt/osmotic tolerance genes [70]. Therefore, similar systems may exist in most thermophilic cyanobacteria studied as responses to temperature upshift and changes in light quality. The homologs of nblS (Synpcc7942_924) and nblR (Synpcc7942_2305) in PCC 7942 were detected in all of the thermophilic cyanobacteria studied (Figures S3 and S4), suggesting the essential role of this system to cope with high light intensity by controlling the light intensity-mediated expression of the hliA gene and several other photosynthesis-related genes, e.g., psbA genes and cpcAB genes [72,73]. Another TCS homologous to sphS (Synpcc7942_1011) and sphR (Synpcc7942_1012) in PCC 7942 was present in all the thermophilic cyanobacteria studied (Figures S3 and S4). This system may be helpful for thermophiles to respond to the phosphate limitation in hot springs [74]. Moreover, homologs of Rre37 (Synpcc6803_sll1330) were found in all the thermophilic cyanobacteria studied, suggesting a potential regulatory network of activating the transcript accumulation of sugar catabolic genes during nitrogen starvation [75]. Taken together, TCS-modulated responses to temperature shift and nutrient deprivation might be crucial for thermophiles to survive and even thrive in thermal environments.

## 4. Conclusions

In the current study, we conducted a comprehensive comparative analysis of the components and structures of TCS in 17 thermophilic cyanobacteria. The results revealed a fascinating complexity and diversity of the TCSs. A distinct composition of the TCS genes existed among these thermophilic cyanobacteria. The TCS genes were mainly organized as orphans, and a high proportion of HKs were predicted to be soluble. The domain architectures of HK and RRs were diversified among these thermophilic cyanobacteria, suggesting their various roles in biological functions. Comparative and evolutionary genomic analyses indicated that HGT, as well as duplications events, might be involved in the evolutionary history of TCS genes in *Thermostichus* and *Thermosynechococcus* strains. A comparative analysis between thermophilic and mesophilic cyanobacteria indicated that one HK cluster and one RR cluster were uniquely shared by all the thermophilic cyanobacteria studied, while two HK clusters and one RR cluster were only common to all the filamentous thermophilic cyanobacteria. These results suggested that these thermophile-unique clusters may be related to thermal characters and the morphology. Overall, these findings provided insights into the TCS of thermophilic cyanobacteria and fundamental knowledge for further research regarding thermophilic cyanobacteria with a broad potential for acclimations to environmental fluctuations.

## Figures and Tables

**Table 1:** Summary of putative TCS genes in thermophilic cyanobacteria studied.

Species	Genome Size (M)	No. of CDS	HK				RR	PP	
			Classic	Hybrid	Unorthodox	CheA		HisKa	Hpt
*Leptodesmis sichuanensis* A121	5.35	4917	42	23	1	2	61	1	1
*Leptolyngbya* sp. JSC-1	7.87	7423	69	49	1	6	111	3	0
*Leptothermofonsia sichuanensis* E412	6.43	5495	60	30	2	3	79	0	1
*Thermoleptolyngbya sichuanensis* A183	5.53	4541	51	15	1	2	46	0	0
*Thermoleptolyngbya* sp. O-77	5.48	4865	53	14	0	2	49	2	0
*Thermostichus* sp. 60AY4M2	3.16	2622	16	1	1	2	29	2	0
*Thermostichus* sp. 63AY4M2	3.09	2577	15	0	2	2	30	2	0
*Thermostichus* sp. 65AY6A5	2.98	2597	17	1	1	2	28	2	0
*Thermostichus* sp. 65AY6Li	2.93	2632	15	1	2	2	29	2	0
*Thermostichus* sp. JA-2-3Ba	3.05	2862	16	1	2	2	28	2	0
*Thermostichus* sp. JA-3-3Ab	2.93	2760	15	1	2	2	29	2	0
*Thermosynechococcus lividus* PCC 6715	2.66	2465	8	3	0	2	32	2	2
*Thermosynechococcus* sp. CL-1	2.65	2549	13	4	0	2	26	1	1
*Thermosynechococcus* sp. TA-1	2.66	2556	12	4	0	2	26	1	2
*Thermosynechococcus vestitus* BP-1	2.59	2475	10	4	0	3	23	1	1
*Thermosynechococcus vestitus* E542	2.65	2543	10	3	0	3	26	1	1
*Thermosynechococcus vulcanus* NIES-2134	2.57	2496	10	4	0	2	24	1	1

**Table 2:** Genetic organizations of TCS genes in thermophilic and reference cyanobacteria.

Species	Orphan	Pair	Triad	Tetrad	Pentad	Total
*Leptodesmis* A121	92	12	5			131
*Leptolyngbya* JSC-1	137	35	9		1	239
*Leptothermofonsia* E412	120	17	4	1	1	175
*Thermoleptolyngbya* A183	91	8	1		1	115
*Thermoleptolyngbya* O-77	84	14	1		1	120
*Thermostichus* 60AY4M2	46	1	1			51
*Thermostichus* 63AY4M2	46	1	1			51
*Thermostichus* 65AY6A5	46	1	1			51
*Thermostichus* 65AY6Li	44	2	1			51
*Thermostichus* JA-2-3Ba	39	3	2			51
*Thermostichus* JA-3-3Ab	42	3	1			51
*Thermosynechococcus* PCC 6715	42	2	1			49
*Thermosynechococcus* CL-1	33	7				47
*Thermosynechococcus* TA-1	35	6				47
*Thermosynechococcus* BP-1	32	5				42
*Thermosynechococcus* E542	38	3				44
*Thermosynechococcus* NIES-2134	36	3				42
*Cylindrospermum stagnale* PCC 7417	107	27	3	1		174
*Microcystis aeruginosa* NIES-843	41	2				45
*Nostoc sp.* PCC 7524	109	25	4			171
*Parasynechococcus* sp. WH7803	19	2	1			26
*Synechocystis* sp. PCC 6803	70	10				90

**Table 3:** Sensing domains detected in HKs of thermophilic cyanobacteria studied. Number before and within brackets referred to the number of corresponding categories in cHK and hyHK, respectively.

	A121	JSC-1	E412	A183	O-77	60AY4M2	63AY4M2	65AY6A5	65AY6Li	JA-2-3Ba	JA-3-3Ab	PCC 6715	CL-1	TA-1	BP-1	E542	NIES-2134	Putative Signals Detected
PAS/PAC	33/0 (32/1)	31/0 (61/1)	47/0 (24/0)	52/0 (17/0)	57/0 (6/0)	22/1 (0/0)	17/1 (0/0)	22/1 (0/0)	17/1 (0/0)	16/0 (0/0)	16/2 (0/0)	3/0 (3/0)	4/0 (2/1)	4/0 (2/1)	4/0 (2/1)	3/0 (1/0)	4/0 (2/1)	Small molecules, ions, gases, light, and redox State sensing
HAMP	7(2)	18(20)	12(3)	8(2)	9(2)	1(0)	1(0)	1(0)	1(0)	1(0)	1(0)	1(0)	1(1)	1(1)	1(1)	1(1)	1(1)	Signal transduction
GAF	20(13)	22(20)	30(9)	31(9)	29(8)	16(0)	14(0)	16(0)	14(0)	9(0)	14(0)	4(4)	5(5)	5(5)	4(5)	5(2)	4(5)	Redox/oxygen sensing; cGMP binding
PHY	2(0)	4(0)	2(0)	0(0)	0(0)	1(0)	1(0)	1(0)	1(0)	0(0)	1(0)	0(0)	0(0)	0(0)	0(0)	0(0)	0(0)	Tetrapyrroles; light-sensing
CHASE	3(2)	7(1)	1(3)	3(0)	3(0)	0(0)	0(0)	0(0)	0(0)	0(0)	0(0)	0(0)	2(0)	2(0)	0(0)	1(0)	0(0)	Small molecules recognition
MASE	0(0)	1(0)	1(0)	0(0)	0(0)	0(0)	0(0)	0(0)	0(0)	0(0)	0(0)	0(0)	0(0)	0(0)	0(0)	0(0)	0(0)	Membrane associated sensor
Cache	2(1)	6(7)	1(2)	1(1)	2(1)	0(0)	0(0)	0(0)	0(0)	0(0)	0(0)	0(0)	0(1)	0(1)	0(1)	0(1)	0(1)	Small molecules recognition
cNMP_binding	0(0)	1(0)	0(0)	0(0)	0(0)	0(0)	0(0)	0(0)	0(0)	0(0)	0(0)	0(0)	0(0)	0(0)	0(0)	0(0)	0(0)	Cyclic nucleotide monophosphate-binding
S_TKc	1(0)	2(0)	0(0)	0(0)	0(0)	0(0)	0(0)	0(0)	0(0)	0(0)	0(0)	0(0)	0(0)	0(0)	0(0)	0(0)	0(0)	Serine/Threonine kinase catalytic domain
NIT	0(0)	0(0)	0(1)	0(1)	0(1)	0(0)	0(0)	0(0)	0(0)	0(0)	0(0)	0(0)	0(0)	0(0)	0(0)	0(0)	0(0)	Nitrate and nitrite responsive
FHA	0(0)	0(0)	0(0)	0(0)	0(0)	0(0)	0(0)	0(0)	0(0)	0(0)	0(0)	0(0)	1(0)	1(0)	1(0)	1(0)	1(0)	Phosphoserine/threonine binding
CBS	3(0)	0(5)	0(2)	1(0)	1(0)	0(0)	0(0)	0(0)	0(0)	0(0)	0(0)	0(0)	0(0)	0(0)	0(0)	1(0)	0(0)	Adenosine nucleotides binding
Pkinase	0(0)	0(1)	0(0)	0(0)	0(0)	0(0)	0(0)	0(0)	0(0)	0(0)	0(0)	0(0)	0(0)	0(0)	0(0)	0(0)	0(0)	ATP binding

**Table 4:** Domain architecture of HKs in thermophilic cyanobacteria studied. Only the top 10 abundant domain architectures of HKs were shown. The full version was summarized in Table S4.

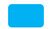 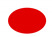 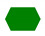 Sensing Transmit Receiver	A121	JSC-1	E412	A183	O-77	60AY4M2	63AY4M2	65AY6A5	65AY6Li	JA-2-3Ba	JA-3-3Ab	PCC 6715	CL-1	TA-1	BP-1	E542	NIES-2134
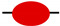	12	16	21	12	14	4	4	5	4	7	4	3	5	4	5	3	5
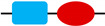	12	27	14	17	16	3	3	3	3	2	3	3	5	5	2	4	2
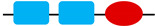	7	16	12	9	10	2	2	2	2	4	2	1	1	1	1	1	1
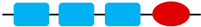	5	7	3	3	3	1	1	1	1	0	1	1	2	2	2	2	2
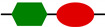	7	9	7	4	4	1	1	1	1	1	1	0	0	0	0	0	0
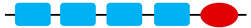	3	0	4	3	2	3	3	3	3	1	3	0	0	0	0	0	0
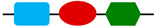	1	5	4	1	1	0	0	0	0	0	0	1	1	1	1	1	1
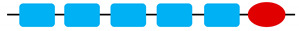	1	1	1	3	3	1	1	1	1	1	1	0	0	0	0	0	0
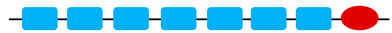	0	1	2	1	3	2	1	2	1	1	0	0	0	0	0	0	0
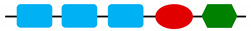	2	2	1	0	0	0	0	0	0	0	0	0	1	1	1	1	1

**Table 5:** Output domains of RRs in thermophilic cyanobacteria studied.

Output Domain	RR Family	A121	JSC-1	E412	A183	O-77	60AY4M2	63AY4M2	65AY6A5	65AY6Li	JA-2-3Ba	JA-3-3Ab	PCC 6715	CL-1	TA-1	BP-1	E542	NIES-2134
Stand-alone REC	CheY	25	47	31	14	12	9	9	9	8	6	7	10	5	5	5	7	6
DNA-binding	NarL	12	12	8	8	10	6	6	6	6	6	6	3	4	4	4	4	4
	OmpR	14	27	24	13	13	7	8	7	8	8	8	7	11	10	7	7	7
Chemotaxis	CheB	0	0	1	0	0	0	0	0	0	0	0	1	0	0	0	0	0
c-di-GMP signaling	RpfG	0	1	1	1	1	1	1	1	1	2	1	0	0	0	0	0	0
	PleD	1	3	4	3	3	2	2	2	2	2	2	2	1	1	1	1	1
	PleD-VieA	1	2	1	1	1	0	0	0	0	0	0	1	0	1	0	0	0
Ser/Thr phosphorylation	RsbU	2	2	2	2	2	1	1	1	1	1	1	1	1	1	1	1	1
Pyridine nucleotide-disulphide oxidoreductase	TrxB	0	1	0	0	0	0	0	0	0	0	0	0	0	0	0	0	0
Carbohydrate utilization	YesN	0	1	1	0	0	0	0	0	0	0	0	0	0	0	0	0	0
Adenylate and Guanylate cyclase catalytic domain	CyC-C	1	1	2	1	1	0	0	0	0	0	0	0	0	0	0	0	0
Unknown	Unclassified	5	13	4	3	6	3	3	2	3	3	4	7	4	4	5	6	5
Total		61	111	79	46	49	29	30	28	29	28	29	32	26	26	23	26	24

## Data Availability

The data presented in this study are openly available in the National Center for Biotechnology Information (https://www.ncbi.nlm.nih.gov/genome/, accessed on 1 January 2022).

## Supplementary Materials

The following supporting information can be downloaded at: https://www.mdpi.com/article/10.3390/biology12020271/s1, Figure S1: ML phylogram of HKs and RRs in *Thermostichus* genomes; Figure S2: ML phylogram of HKs and RRs in *Thermosynechococcus* genomes; Figure S3: NJ phylogram of HKs representing the thermophiles studied and mesophilic *Synechococcus* PCC 7942 and *Synechocystis* PCC 6803; Figure S4: NJ phylogram of RRs representing the thermophiles studied and mesophilic *Synechococcus* PCC 7942 and *Synechocystis* PCC 6803; Table S1: Morphology, ecological information and genome characteristics of thermophilic cyanobacteria studied; Table S2: Predicted TCS genes in the genomes of thermophilic cyanobacteria studied; Table S3: Habitat and number of TCS genes of cyanobacteria studied; Table S4: Number of domain architecture of HKs in thermophilic cyanobacteria studied; Table S5: Ortholog tables of HKs and RRs in *Thermostichus* and *Thermosynechococcus* strains.
