# Supplementary material for: Distinct Molecular Patterns of Two-Component Signal Transduction Systems in Thermophilic Cyanobacteria as Revealed by Genomic Identification
Source: Biology (Basel). 2023 Feb 8;12(2):271. doi: 10.3390/biology12020271 (PMC9953108; doi:10.3390/biology12020271)
Supplement: Supplementary file 1 [file biology-12-00271-s001.zip › Fig S1.pdf]

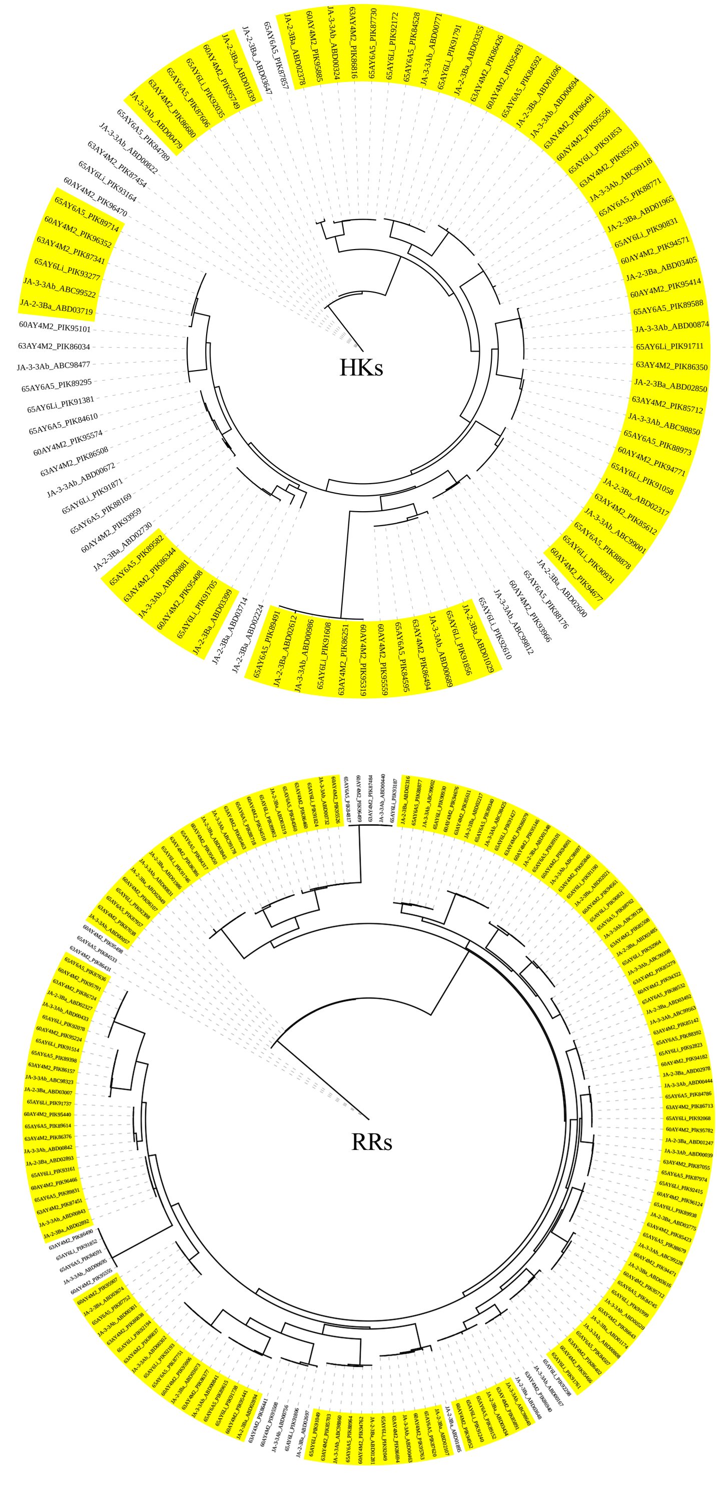


**Figure S1** ML phylogram of HKs and RRs in *Thermostichus* genomes. Orthologous genes shared by all strains were highlighted in yellow.
