# Supplementary material for: Distinct Molecular Patterns of Two-Component Signal Transduction Systems in Thermophilic Cyanobacteria as Revealed by Genomic Identification
Source: Biology (Basel). 2023 Feb 8;12(2):271. doi: 10.3390/biology12020271 (PMC9953108; doi:10.3390/biology12020271)
Supplement: Supplementary file 1 [file biology-12-00271-s001.zip › Fig S3.pdf]

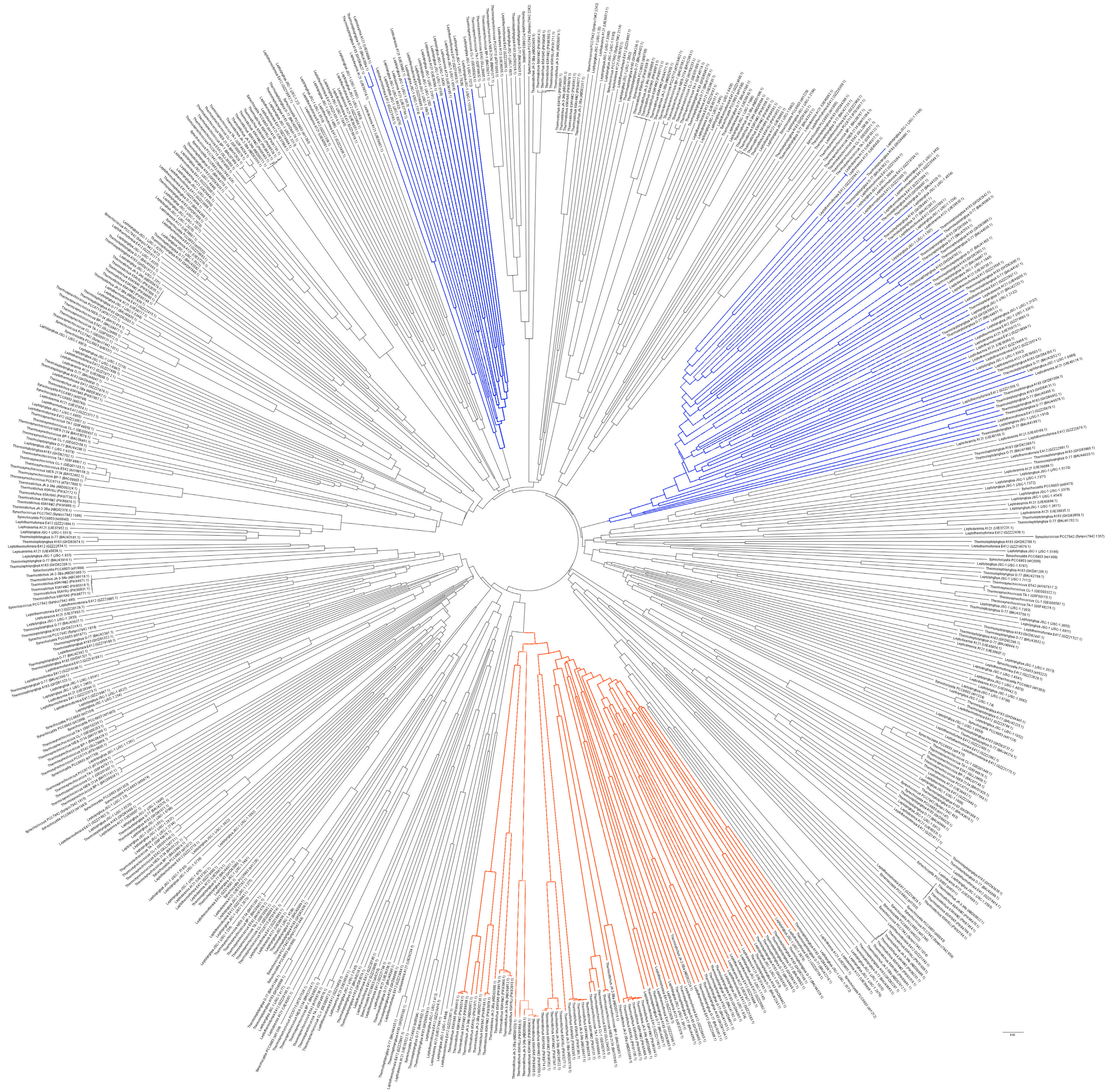

Figure S3 NJ phylogram of HKs representing the thermophiles studied and mesophilic *Synechococcus* PCC 7942 and *Synechocystis* PCC 6803. Red branches indicates the HKs that are uniquely shared by all the thermophilic cyanobacteria studied, while blue branches indicates the HKs that are only common to all the filamentous thermophilic cyanobacteria.
