# Supplementary material for: Distinct Molecular Patterns of Two-Component Signal Transduction Systems in Thermophilic Cyanobacteria as Revealed by Genomic Identification
Source: Biology (Basel). 2023 Feb 8;12(2):271. doi: 10.3390/biology12020271 (PMC9953108; doi:10.3390/biology12020271)
Supplement: Supplementary file 1 [file biology-12-00271-s001.zip › Fig S4.pdf]

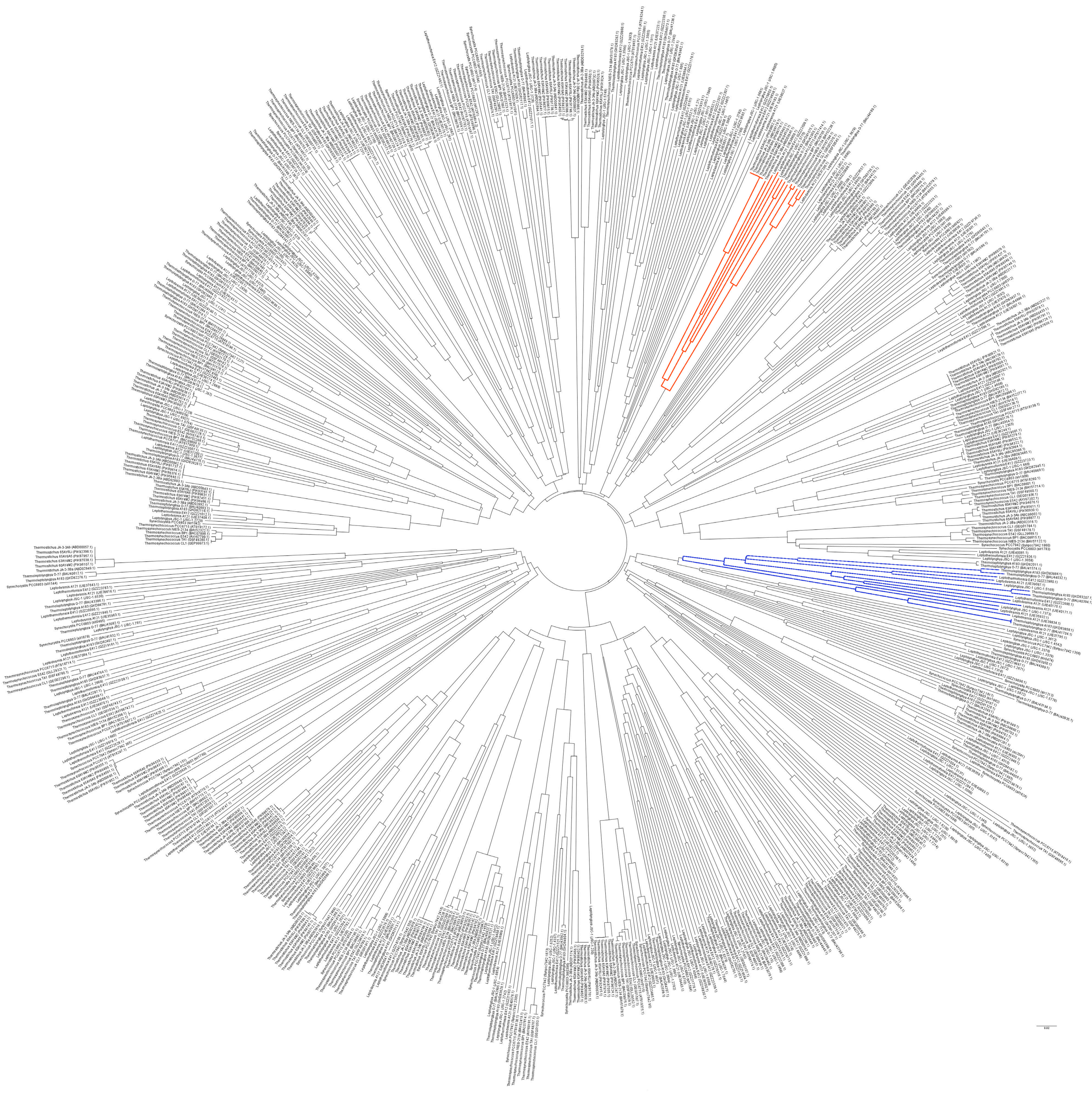

Figure S4 NJ phylogram of RRs representing the thermophiles studied and mesophilic *Synechococcus* PCC 7942 and *Synechocystis* PCC 6803. Red branches indicates the RRs that are uniquely shared by all the thermophilic cyanobacteria studied, while blue branches indicates the RRs that are only common to all the filamentous thermophilic cyanobacteria.
